# Macrophage CD74 contributes to MIF-induced pulmonary inflammation

**DOI:** 10.1186/1465-9921-10-33

**Published:** 2009-05-04

**Authors:** Koichiro Takahashi, Kiyokazu Koga, Helena M Linge, Yinzhong Zhang, Xinchun Lin, Christine N Metz, Yousef Al-Abed, Kaie Ojamaa, Edmund J Miller

**Affiliations:** 1 Center for Heart and Lung Research, The Feinstein Institute for Medical Research, Manhasset, New York, USA; 2 Medicinal Biochemistry, The Feinstein Institute for Medical Research, Manhasset, New York, USA; 3 Medicinal Chemistry, The Feinstein Institute for Medical Research, Manhasset, New York, USA; 4 Molecular Cardiovascular Research, The Feinstein Institute for Medical Research, Manhasset, New York, USA; 5 Departments of Medicine and Surgery, Albert Einstein College of Medicine, Bronx, New York, USA

## Abstract

**Background:**

MIF is a critical mediator of the host defense, and is involved in both acute and chronic responses in the lung. Neutralization of MIF reduces neutrophil accumulation into the lung in animal models. We hypothesized that MIF, in the alveolar space, promotes neutrophil accumulation via activation of the CD74 receptor on macrophages.

**Methods:**

To determine whether macrophage CD74 surface expression contributes MIF-induced neutrophil accumulation, we instilled recombinant MIF (r-MIF) into the trachea of mice in the presence or absence of anti-CD74 antibody or the MIF specific inhibitor, ISO-1. Using macrophage culture, we examined the downstream pathways of MIF-induced activation that lead to neutrophil accumulation.

**Results:**

Intratracheal instillation of r-MIF increased the number of neutrophils as well as the concentration of macrophage inflammatory protein 2 (MIP-2) and keratinocyte-derived chemokine (KC) in BAL fluids. CD74 was found to be expressed on the surface of alveolar macrophages, and MIF-induced MIP-2 accumulation was dependent on p44/p42 MAPK in macrophages. Anti-CD74 antibody inhibited MIF-induced p44/p42 MAPK phosphorylation and MIP-2 release by macrophages. Furthermore, we show that anti-CD74 antibody inhibits MIF-induced alveolar accumulation of MIP-2 (control IgG vs. CD74 Ab; 477.1 ± 136.7 vs. 242.2 ± 102.2 pg/ml, p < 0.05), KC (1796.2 ± 436.1 vs. 1138.2 ± 310.2 pg/ml, p < 0.05) and neutrophils (total number of neutrophils, 3.33 ± 0.93 × 10^4 ^vs. 1.90 ± 0.61 × 10^4^, p < 0.05) in our mouse model.

**Conclusion:**

MIF-induced neutrophil accumulation in the alveolar space results from interaction with CD74 expressed on the surface of alveolar macrophage cells. This interaction induces p44/p42 MAPK activation and chemokine release. The data suggest that MIF and its receptor, CD74, may be useful targets to reduce neutrophilic lung inflammation, and acute lung injury.

## Background

Macrophage migration inhibitory factor (MIF) is an inflammatory mediator of innate and adaptive immune responses. MIF protein is present in most cells including pituitary cells, T cells, macrophages/monocytes, and is released in response to infection and stress [1]. Plasma MIF concentrations are elevated in patients with inflammatory diseases such as sepsis [2], ARDS [3,4] or rheumatoid arthritis [5]. In addition, plasma concentration of MIF is positively correlated with the severity of sepsis [6,7]. Furthermore, mice deficient in the MIF gene, or those in which the MIF protein has been neutralized, are protected from lethal endotoxemia and septic shock [8,9].

In lung inflammation and injury models, MIP-2 and KC play key roles in neutrophil accumulation into the lungs [10]. Neutrophils are an important component of the inflammatory response in acute lung injury [11,12]. The elimination of neutrophils can markedly decrease the severity of lung injury in animal lung inflammation models [13]. MIF levels are increased in BAL fluid in lipopolysaccharide (LPS)-induced lung injury model [14], and MIF neutralizing antibody blocks LPS-induced pulmonary neutrophil accumulation in animal models [15,16], suggesting that MIF can influence neutrophil accumulation into the lungs.

CD74 (also known as a MHC class II invariant chain) is a type II transmembrane protein, reported to be part of the MIF receptor complex [17]. Several studies have shown that CD74 is expressed both intracellularly and on the cell surface in B cell lymphoma [18], T cell lymphoma, melanoma cells [19] and gastric epithelial cells [20]. MIF binds to cell surface CD74, and induces p44/p42 MAPK phosphorylation and cell proliferation. Moreover neutralization of CD74 inhibits MIF-induced cell proliferation in B cells and fibroblasts [17]. Other recent studies have shown that anti-CD74 antibody blocks MIF-CD74 binding on the cell surface of gastric epithelial cells [21], and anti-CD74 antibody attenuated proliferation of prostate cancer cells [22]. CD74 has a short N-terminal cytoplasmic domain of 28 amino acids and appears to lack intracellular signaling domains. Recently, CD44 has been identified as an accessory protein required for MIF-CD74 signal transduction [23].

MIF is found at increased levels in BAL fluids from both LPS induced lung inflammation [14] and polymicrobial sepsis models [24]. BAL fluid-MIF levels in ARDS patients were also significantly increased compared with healthy controls [3]. However, little is known about the mechanisms involved in MIF-induced lung inflammation. We have previously shown that MIF itself causes neutrophil accumulation into the alveolar space [25]. MIF is an intracellular protein that can be released into the extracellular environment where it acts as a potent inflammatory stimulant. Extracellular MIF can bind to the cell surface molecule CD74.

Therefore we focused on the MIF receptor CD74 in an animal model. We hypothesized that MIF, in the alveolar space, results in neutrophil accumulation via activation of the CD74. Here we used intra-tracheal instillation of MIF, and studied the contribution of CD74 in MIF-induced neutrophil accumulation in a mouse model.

The present study demonstrated that MIF instillation increased the concentration of MIP-2 and KC as well as the number of neutrophils in the alveolar space. This study shows that CD74 expressed on the cell surface of alveolar macrophages, contributes to the MIF-induced neutrophil accumulation into the alveolar space.

## Methods

### Mouse model

All experiments were approved by the Institutional Animal Care and Use Committee at The Feinstein Institute for Medical Research at North Shore-Long Island Jewish Health System. C57Bl/6 male mice (8–12 weeks old) were anesthetized with isoflurane and the trachea was surgically exposed. Recombinant murine MIF (r-MIF; 1.0 μg in 50 μl normal saline) or 50 μl normal saline alone (control) was instilled directly into the lungs via the trachea using a 29 gauge needle. Groups of mice (n = 6/group) were euthanized at 3, 6 and 20 hours post instillation, blood was collected by cardiac puncture. The blood samples were centrifuged (2000 × g, 10 min), and the plasma was stored at -80°C for further examination. Postmortem bronchoalveolar lavage (BAL) was performed by instilling and withdrawing sterile physiological saline (1 ml) through a tracheal cannula using a 20 gauge Surflo i.v. catheter (Terumo, Elkton, MD). This procedure was repeated three times, and the three BAL fluid samples were pooled. Lung tissues were then isolated and frozen immediately in liquid nitrogen. The BAL fluid was centrifuged (300 × g, 5 min), and the supernatant portions were stored at -80°C for further examination.

### Differential cell count in BAL fluid

Immediately after collection of BAL fluids, erythrocytes were lysed using 0.2% saline and the remaining cells were resuspended in Hanks' Balanced Salt Solution (Invitrogen, Grand Island, NY). Total cell count of each BAL sample was determined using a Neubauer hemocytometer (Hausser Scientific, Horsham, PA). Differential cell counts were performed (200 cells for each experimental condition) on cytospin slides stained with Protocol HEMA3 solution (Fisher scientific, Fair Lawn, NJ). Total protein concentration in BAL fluid was measured using Coomassie protein assay kit (Thermo Scientific, Rockford, IL).

### Reagents and antibodies

r-MIF was prepared from an Escherichia coli expression system, and treated with polymyxin B as previously described [26]. The MIF specific inhibitor (S, R)-3-(4-hydroxyphenyl)-4,5-dihydro-5-isoxazole acetic acid methyl ester (ISO-1) was diluted for use with a minimal amount of dimethyl sulfoxide (DMSO, Fisher Scientific, Fair Lawn, NJ) as previously described [24,27,28]. Anti-CD74 antibody (clone; In-1) for western blotting was purchased from BD Bioscience (San Jose, CA), and anti-CD74 goat polyclonal antibody for flow cytometry and immunofluorescence was purchased from Santa Cruz Biotechnology Inc. (Santa Cruz, CA). Anti-phospho p44/p42 MAPK, anti-phospho p38 MAPK, anti-p38 MAPK, anti-phospho JNK, anti-JNK and anti-GAPDH antibodies were purchased from Cell Signaling Technology (Danvers, MA). Stripping buffer for membrane was purchased from Thermo Scientific (Rockford, IL). Anti-p44/p42 MAPK antibody and isotype control goat IgG were purchased from Santa Cruz Biotechnology Inc. MAPK specific inhibitor PD98059 and p38 MAPK specific inhibitor SB202190 were purchased from Calbiochem (San Diego, CA).

### Cell culture

Murine macrophages (RAW264.7) and murine alveolar type II epithelial cells (MLE-12) were purchased from American Type Culture Collection (Manassas, VA). RAW264.7 cells were cultured in DMEM supplemented with 10% heat-inactivated fetal calf serum (FCS), L-glutamine, penicillin and streptomycin (Invitrogen, Grand Island, NY) at 37°C in a 5% CO_2 _humidified incubator. MLE-12 cells were cultured in DMEM/F12 media supplemented with 2% FCS, 15 mM HEPES, L-glutamine, penicillin and streptomycin [29,30]. In experiments of anti-CD74 antibody and ISO-1 treatment in vitro, RAW264.7 cells were treated with 10 μg/ml anti-CD74 antibody or 10 μg/ml control goat IgG at 37°C for 30 min. After 30 min pre-treatment, cells were stimulated with 100 ng/ml MIF at 37°C for 10 min. In addition, 100 mM ISO-1 in PBS or 5% DMSO in PBS and 100 ng/ml MIF were mixed in microtubes at 37°C for 30 min, then cells were stimulated with the mixture at 37°C for 10 min. Cells were lysed and subjected to SDS-PAGE, then immunoprobed by anti-phospho and total p44/p42 MAPK antibody.

### Western blot analysis

A total of 1.0 × 10^6 ^RAW264.7 cells/sample were stimulated with various concentrations of r-MIF in 1% FCS containing media. After washing (×2) with ice cold PBS buffer, cells were lysed in lysis buffer containing 150 mM NaCl, 50 mM Tris-HCl (pH 7.4), 2 mM EDTA (pH 8.0), 1% octylphenol polyethyleneglycol (NP-40), 0.5% deoxycholic acid, 0.1% SDS, and 1 mM PMSF. Lysates were separated from debris by centrifugation (7800 × g) for 15 min, and lysates were boiled for 5 min in Laemmli sample buffer (Bio-Rad Laboratories, Hercules, CA) under reducing conditions. Tissues were thawed and homogenized in lysis buffer at 4°C, and incubated on ice for 30 min, then centrifuged (7800 × g) for 15 min at 4°C. The supernatants were analyzed for protein content, and were boiled for 5 min in Laemmli sample buffer (Bio-Rad Laboratories). Proteins (total protein content was 15 μg/lane) were separated by sodium dodecyl sulfate polyacrylamide gel electrophoresis (SDS-PAGE), and transferred to a polyvinylidine difluoride membrane (Millipore, Billerica, MA). Prestained molecular weight standards (Crystalgen Inc., Plainview, NY) were run with each gel to determine the approximate molecular weight of detected bands. Then membranes were incubated with the specific primary antibodies and horse radish peroxidase (HRP) conjugated secondary antibodies. After washing with tris-buffered saline contained 0.1% tween-20 (TBS-T), membranes were incubated with Chemiluminescence Luminol Reagent (Amersham Biosciences, Pittsburgh, PA) and exposed to photographic film (Fujifilm Corp, Tokyo, Japan). Protein bands were quantified by densitometric analysis using a Gel Doc 2000 Chemi Doc scanner (Bio-Rad Laboratories) and Quantity One 4.4.0 software.

### Cell staining and flow cytometry

Single-cell suspensions were incubated at 4°C for 2 hours with anti-CD74 antibody or control IgG diluted in staining buffer (PBS containing 2% FCS). Cells were washed with staining buffer, and then incubated at 4°C for 30 min with FITC-conjugated secondary antibody. Flow cytometry analysis was performed on FACSCalibur (BD Biosciences, San Jose, CA) and data were analyzed using CellQuest software.

### Cytokine analysis

Cytokine concentrations were evaluated using commercially available enzyme-linked immunosorbent assay (ELISA) kits for keratinocyte-derived chemokine (KC; CXCL1) and macrophage inflammatory protein 2 (MIP-2; CXCL2) from R&D Systems. (Minneapolis, MN).

### Histological study

To obtain lungs for routine histology, the trachea was cannulated and the lungs were gently fixed at inflation with 4% paraformaldehyde solution. The fixed lungs were sectioned (5 μm), and the sections stained with hemotoxylin and eosin (Sigma-Aldrich, St. Louis, MO). For identification of neutrophils, the sections were stained with naphthol AS-D chloroacetate esterase (Sigma-Aldrich, St. Louis, MO).

### Immunofluorescence study

Cells from BAL fluid were prepared using cytospin centrifugation. The slides were fixed in 4% paraformaldehyde in PBS, pH 7.4 for 10 min at room temperature (RT) and permeabilized with 0.01% Triton X-100 in PBS for 5 min at RT. After washing with PBS, each slide was blocked with 10% rabbit serum in PBS for 1 hour at RT. Slides were then incubated with anti-CD74 polyclonal antibody or control IgG diluted in blocking solution for 18 hours at 4°C, and were incubated with FITC-conjugated secondary antibody diluted in blocking solution for 1 hour at RT. After washing with PBS, slides were incubated with 4', 6-diamidino-2-phenylindole, dihydrochloride (DAPI, AnaSpec Inc. San Jose, CA) in blocking solution for 30 min at RT. Cover slips were then placed on the slides with mounting medium (Vector laboratories, Inc. Burlingame, CA). Images were obtained using a Zeiss ApoTome fluorescent microscope and Zeiss Axiovision software (Zeiss LSM, Thornwood, NY).

### Statistical analysis

All values are expressed as mean ± standard deviation (SD). Multiple groups were compared using analysis of variance (ANOVA) using the Dunnett's post hoc test. Results were considered statistically significant at p < 0.05.

## Results

### MIF induces neutrophil accumulation into the lung

**Figure 1:**
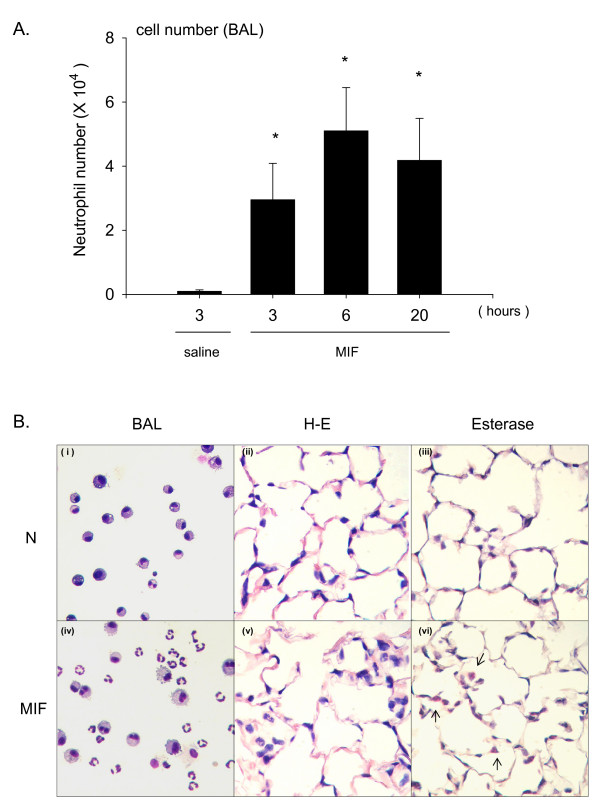
**MIF induces neutrophil accumulation into the alveolar space**. Mice were euthanized at 3, 6 or 20 hours post instillation of 1.0 μg MIF or 3 hours (saline controls). Lungs were lavaged three times with normal saline. A. The number of neutrophil in the bronchoalveolar lavage (BAL) fluid was counted by hemocytometer. Bars indicate mean ± SD (n = 6/group). *P < 0.05 compared with normal saline control. B. (i), (iv) show the cell population in BAL fluid with HEMA3 stain. (i)-(iii) shows representative samples from a control mouse, and (iv)-(vi) shows samples from 6 hours after MIF instilled mice. Lung tissue stained with H-E stain in (ii), (v) and naphthol AS-D chloroacetate esterase stain (neutrophil granules were stained with bright red color, point with arrows) in (iii), (vi).

**Figure 2:**
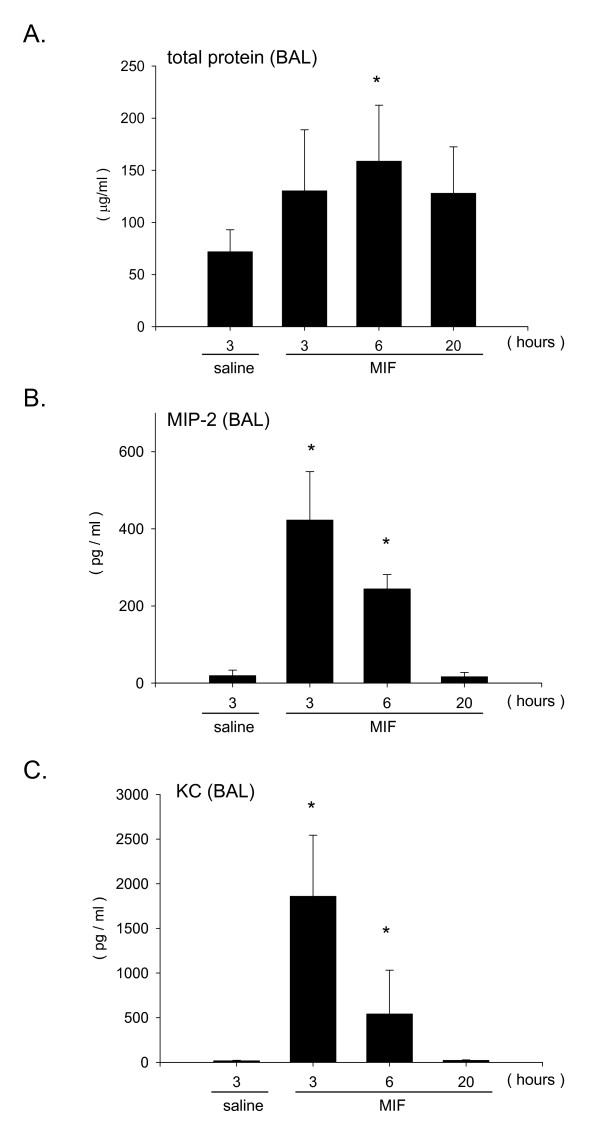
**Alveolar protein and chemokine response following intratracheal instillation of MIF**. Mice were euthanized at 3, 6 or 20 hours post instillation of MIF, and the lungs were lavaged with normal saline. A. Total protein concentration in BAL fluid after 3, 6, 20 hours. B. MIP-2 concentration in BAL fluid after 3, 6, 20 hours measured by ELISA. C. KC concentration in BAL fluid measured by ELISA. Bars indicate mean ± SD (n = 6/group). *P < 0.05 compared with normal saline control.

We have previously reported that MIF has the ability to induce neutrophil accumulation into the alveolar space of mice [25]. To examine the kinetics of MIF-induced leukocytosis, C57Bl/6 mice were instilled intratracheally with r-MIF and the influx of neutrophils was examined over time. The number of neutrophils in BAL fluid at 3, 6 or 20 hours after instillation of MIF significantly increased compared to control animals in which normal saline was instilled alone (Fig. 1A). The number of neutrophils was highest at 6 hours post instillation (total number of neutrophils, saline; 0.09 ± 0.04 × 10^4^, 3 hours; 2.95 ± 1.13 × 10^4^, 6 hours; 5.10 ± 1.35 × 10^4^, 20 hours; 4.18 ± 1.31 × 10^4^). At 6 hours post instillation neutrophil number increased not only in the BAL fluid (Fig. 1B, i and 1B, iv) but also in the alveolar tissue (Fig. 1B, ii, iii, v, vi). BAL fluids were assessed for total protein content, and the neutrophil chemoattractants MIP-2 and KC. Total protein concentration in BAL fluids significantly increased at 6 hours after MIF instillation (Fig. 2A, saline; 71.9 ± 21.1 μg/ml, 3 hours; 130.4 ± 58.5 μg/ml, 6 hours; 158.7 ± 53.6 μg/ml, 20 hours; 127.9 ± 44.5 μg/ml). Furthermore, MIP-2 and KC in BAL fluids increased at 3, 6 hours after instillation and peaked around 3 hours (Fig. 2B, the concentration of MIP-2 at 3 hours; 422.7 ± 125.7 pg/ml, Fig. 2C, the concentration of KC at 3 hours; 1859.4 ± 684.1 pg/ml). However, MIP-2 and KC concentrations in plasma from these mice did not significantly change following MIF instillation (data not shown).

### Macrophages express MIF receptor component CD74

**Figure 3:**
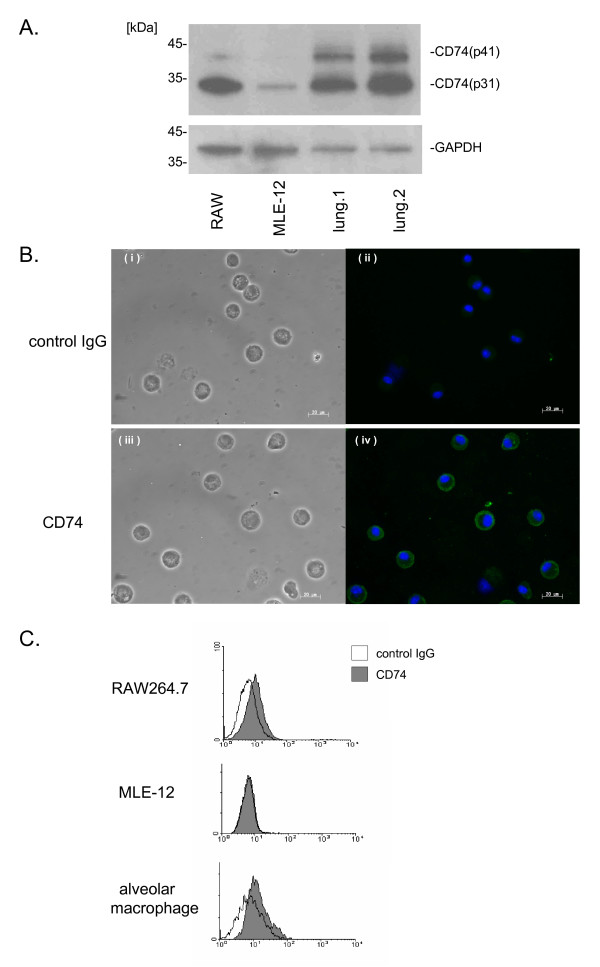
**CD74 expresses on the cell surface of alveolar macrophages**. A. RAW264.7, MLE-12 and lung tissues from two non-treated mice were homogenized in lysis buffer and prepared for western blot analysis for CD74. The blots were then stripped and re-probed for GAPDH antibody as a loading control (total protein content was 15 μg/lane). Data represents three independent experiments. B. BAL cells from mice were stained with anti-CD74 antibody or control IgG and subsequent FITC-conjugated secondary antibody after fixation and permeabilization. (i), (ii) show the control IgG and (iii), (iv) show anti-CD74 antibody. (i), (iii) show differential interference contrast (DIC) and (ii), (iv) show fluorescent image. Green color represents CD74 or control IgG (FITC) and blue color represents nuclear (DAPI). Scale bar represents 20 μm. Data represents four independent experiments. C. Flow cytometry of non-permeabilized RAW264.7, MLE-12 and alveolar macrophages assessing surface expression of CD74. Shaded histograms show CD74 staining, and opened histograms show the control IgG staining. Data shown is representative of three independent experiments.

MIF intratracheal instillation markedly induced neutrophil accumulation in the lung. To determine the involvement of CD74 in the MIF-induced responses, we examined CD74 expression in several cell types. We used RAW264.7 cells and the MLE-12 cells as models of alveolar macrophages and type II alveolar epithelial cells respectively, and assayed for the presence of CD74 in RAW264.7, MLE-12 and lung tissues. Two isoforms of CD74 (p41 and p31) were observed. There was higher expression of CD74 protein in RAW264.7 and lung tissues than in MLE-12 cells (Fig. 3A). To confirm CD74 expression in primary alveolar macrophages in BAL fluid, we used immunofluorescence microscopy. We found that CD74 protein is also expressed in alveolar macrophages (Fig. 3B). More than 95% of the cells in BAL fluid were recognized as macrophages using stained cytospin slides in this experiment. Furthermore, cell surface expression of CD74 was evident in non-permeabilized RAW264.7 and alveolar macrophages but not in MLE-12 (Fig. 3C).

### MIF activates p44/p42 MAPK pathway and stimulates MIP-2 release from macrophages

**Figure 4:**
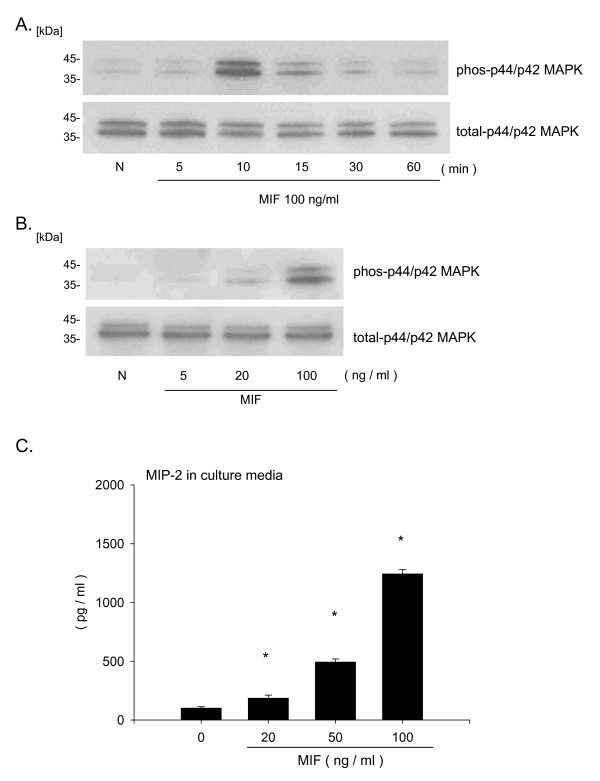
**MIF activates p44/p42 MAPK pathway and stimulates MIP-2 release from macrophages**. A. RAW264.7 cells were stimulated with 100 ng/ml MIF for 5, 10, 15, 30 or 60 min. After stimulation the cells were lysed and boiled for SDS-PAGE analysis. Transferred proteins were detected by anti-phospho and total p44/p42 MAPK antibodies. Data represents four independent experiments. B. RAW264.7 cells were stimulated with 5, 20, 100 ng/ml MIF for 10 min. Proteins were detected by anti-phospho and total p44/p42 MAPK antibodies. Data represents three independent experiments. C. RAW264.7 cells were stimulated with different concentrations of MIF (20, 50, 100 ng/ml) or control media for 18 hours at 37°C. After 18 hours supernatants were collected and MIP-2 concentrations were measured by ELISA. Bars indicate mean ± SD (n = 3/group). Data is representative of four independent experiments. *P < 0.05 compared with control media.

**Figure 5:**
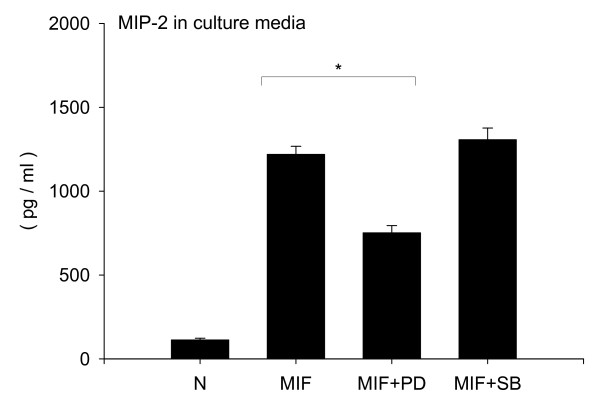
**MIF-induced MIP-2 accumulation depends on p44/p42 MAPK pathway**. RAW264.7 cell were incubated with MAP kinase kinase (MEK) specific inhibitor (PD98059, 30 mM) or p38 MAP kinase specific inhibitor (SB202190, 30 mM) or DMSO for 30 min before stimulation with 100 ng/ml MIF. After 18 hours stimulation, supernatants were collected and MIP-2 concentrations in supernatants were measured by ELISA. Bars indicate mean ± SD (n = 3/group). Data is representative of three independent experiments. *P < 0.05 compared with DMSO + MIF.

Following MIF stimulation, the p44/p42 MAPK signaling pathway was activated in both a time and dose dependent manner in RAW264.7 macrophage cells (Fig. 4A, 4B). However, there was no significant activation of p38 MAPK or JNK signaling pathways (data not shown). To investigate whether MIF stimulation induces the release of neutrophil chemokines, we measured MIP-2 and KC in cell culture supernatants. There was a significant accumulation of MIP-2 (Fig. 4C, MIP-2 at 100 ng/ml stimulation; 1243.1 ± 35.3 pg/ml) but not KC (data not shown) in the culture media following stimulation with MIF. To confirm that activation of MIP-2 is downstream of p44/p42 MAPK following MIF stimulation, specific MAP kinase inhibitors were used. The p44/p42 MAPK specific inhibitor PD98059 attenuated the MIF-induced MIP-2 accumulation (Fig. 5, MIF; 1219.7 ± 48.1 pg/ml, MIF + PD; 752.2 ± 42.6 pg/ml, p < 0.05). However, the p38 MAPK inhibitor SB202190 had no effect on the MIP-2 accumulation (Fig. 5, MIF + SB; 1307.6 ± 68.8 pg/ml). This suggests that MIF-induced MIP-2 accumulation depends, at least in part, on p44/p42 MAPK signaling pathway.

### Anti-CD74 antibody inhibits MIF-induced p44/p42 MAPK phosphorylation and MIP-2 accumulation in macrophages

**Figure 6:**
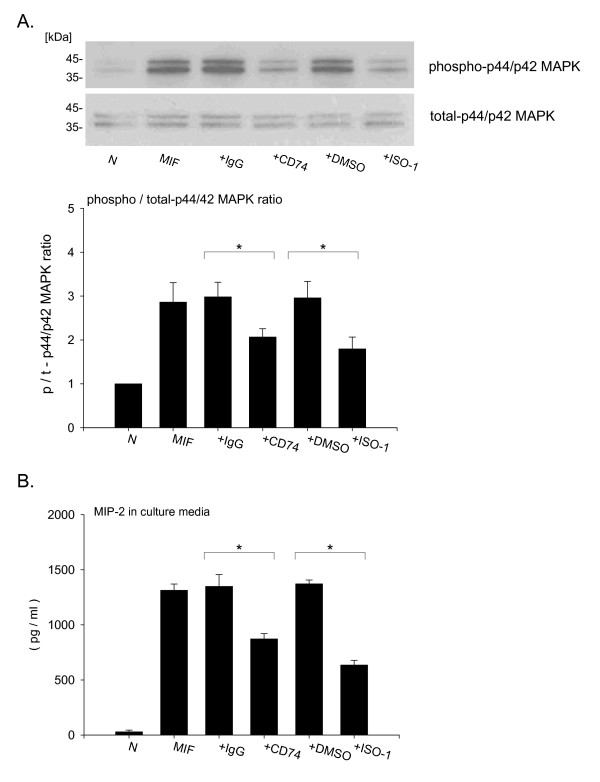
**Anti-CD74 antibody treatment inhibits MIF-induced p44/p42 MAPK phosphorylation and MIP-2 accumulation in macrophages**. A. RAW264.7 cells were treated with 10 μg/ml anti-CD74 antibody or control IgG, or 100 mM ISO-1 or DMSO control for 10 min. Cells were lysed and subjected to SDS-PAGE, immunoprobed by anti-phospho and total p44/p42 MAPK antibody. Bar graphs show the density ratio of phospho- vs. total-p44/p42 MAPK bands. Bar graphs represent the mean ± SD of three independent experiments. B. RAW264.7 cells were treated with 10 μg/ml anti-CD74 antibody or control IgG, or 100 mM ISO-1 or DMSO control for 18 hours. After 18 hours supernatant were collected and MIP-2 concentration were measured by ELISA. Bars indicate mean ± SD (n = 3/group). Data represents four independent experiments. *P < 0.05 compared with control IgG + MIF or DMSO + MIF stimulation.

MIF signaling occurs following MIF binding to CD74. To examine whether neutralization of CD74 can inhibit the MIF-induced cell signaling, we used a CD74 antibody, and the specific MIF inhibitor, ISO-1 [24,27,28]. We found that both CD74 antibody and ISO-1 treatment significantly inhibited MIF-induced phosphorylation of p44/p42 MAPK (Fig. 6A, relative ratio, MIF + control IgG; 2.98 ± 0.33, MIF + CD74 Ab; 2.07 ± 0.19, p < 0.05, MIF + DMSO; 2.96 ± 0.38, MIF + ISO-1; 1.79 ± 0.27, p < 0.05). Furthermore, both ISO-1 and CD74 antibody treatment inhibited MIF-induced MIP-2 accumulation in culture media (Fig. 6B, MIF + control IgG; 1348.7 ± 107.3 pg/ml, MIF + CD74 Ab; 873.0 ± 47.1 pg/ml, p < 0.05, MIF + DMSO; 1372.2 ± 34.6 pg/ml, MIF + ISO-1; 635.7 ± 42.5 pg/ml, p < 0.05). These data suggest that anti-CD74 antibody has a similar effect as ISO-1 treatment in inhibiting MIF-induced cell activation.

### Anti-CD74 antibody inhibits MIF-induced neutrophil accumulation into the alveolar space

**Figure 7:**
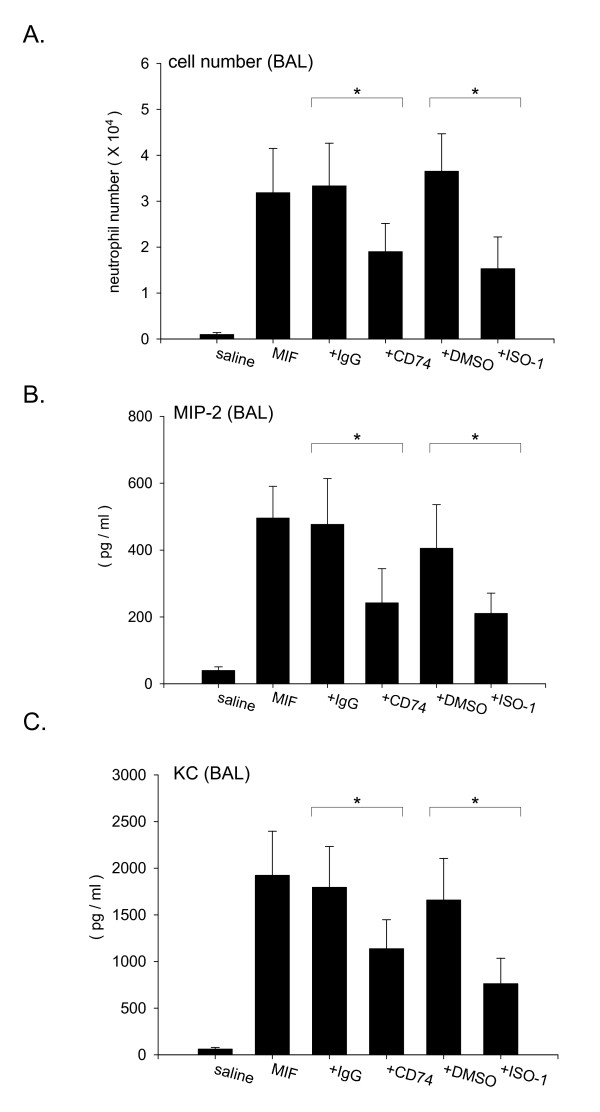
**Anti-CD74 antibody treatment inhibits MIF-induced neutrophil accumulation into the alveolar space**. A. The number of neutrophils in BALF from treated mice. The groups of mice were instilled via the trachea with either 1.0 μg MIF plus 10 μg anti-CD74 antibody or 1.0 μg MIF plus 10 μg control IgG. The other groups of mice were instilled with either 1.0 μg MIF plus 0.5 mg ISO-1 or 1.0 μg MIF plus 5% DMSO. Mice were euthanized 3 hours post instillation, then lungs were lavaged three times with normal saline. The number of neutrophils were counted using a hemocytometer. *P < 0.05 compared with control IgG + MIF or DMSO + MIF stimulation, B. MIP-2, and C. KC concentration in BAL fluid from 3 hours post instilled mice measured by ELISA. Bars indicate mean ± SD (n = 6/group). *P < 0.05 compared with control IgG + MIF or DMSO + MIF stimulation.

In the next series of experiments, MIF was intratracheally instilled in the presence of anti-CD74 antibody or ISO-1, an MIF inhibitor that blocks binding to CD74. Mixtures of either 1.0 μg MIF and 10 μg anti-CD74 antibody or 1.0 μg MIF and 0.5 μg ISO-1 were instilled via the trachea to ensure identical distribution of both agonist and inhibitor. Anti-CD74 antibody and ISO-1 significantly inhibited MIF-induced neutrophil accumulation into the lung (Fig. 7A, total number of neutrophils, MIF + control IgG; 3.33 ± 0.92 × 10^4^, MIF + CD74 Ab; 1.90 ± 0.61 × 10^4^, p < 0.05, MIF + DMSO; 3.65 ± 0.82 × 10^4^, MIF + ISO-1; 1.53 ± 0.69 × 10^4^, p < 0.05). To assess the effect of anti-CD74 antibody treatment on chemokine accumulation, MIP-2 and KC concentrations were measured in the BAL fluids. Anti-CD74 antibody and ISO-1 treatment significantly inhibited the MIF-induced MIP-2 (Fig. 7B, MIF + control IgG; 477.1 ± 136.7 pg/ml, MIF + CD74 Ab; 242.2 ± 102.2 pg/ml, p < 0.05, MIF + DMSO; 405.3 ± 130.5 pg/ml, MIF + ISO-1; 210.6 ± 60.6 pg/ml, p < 0.05) and KC accumulation in BAL fluids (Fig. 7C, MIF + control IgG; 1796.2 ± 436.1 pg/ml, MIF + CD74 Ab; 1138.2 ± 310.3 pg/ml, p < 0.05, MIF + DMSO; 1659.8 ± 444.5 pg/ml, MIF + ISO-1; 763.4 ± 271.4 pg/ml, p < 0.05). Taken together, anti-CD74 antibody and ISO-1 both had an inhibitory effect on MIF-induced MIP-2, KC accumulation and resultant neutrophil accumulation into the alveolar space. These data suggest that CD74 has a pivotal role in MIF-induced neutrophil accumulation into the alveolar space.

## Discussion

MIF is expressed in multiple immune and nonimmune cell types and is released in response to infection and other stresses [1]. MIF exists as a homotrimer, each monomer being approximately 12.5 kDa. MIF has enzymatic activities, and is a potent regulator of innate and adaptive immune responses [1]. MIF has immunoregulatory functions in sepsis [2], ARDS[3], bronchial asthma [31], rheumatoid arthritis [5] and tumorgenesis [32].

Neutrophils play an important role in the inflammatory response, and can be associated with severe lung injury in patients with the acute respiratory distress syndrome (ARDS) [11]. Previous studies suggest that MIF participates in neutrophil accumulation into the lung after intraperitoneal LPS injection [15]. In the LPS intratracheal instillation model, neutralization of MIF attenuated capillary leak and the levels of TNF-α and IL-6 in BAL fluid [14]. The elimination of neutrophils, using anti-neutrophil antibody, markedly decreases the severity of animal acute lung injury in animal models [13]. Neutrophil recruitment from blood into tissue at sites of inflammation usually occurs in post-capillary venules and requires capture, rolling and adhesion on endothelial cells in acute lung injury [12]. A multitude of molecules including selectin, integrin, and immunoglobulin adhesion molecules, cytokines and chemokines participate in this sequential process in a variety of vascular beds [33,34]. The CXC chemokine interleukin-8 (IL-8) has been implicated in mediating the influx of neutrophils into the lung in ARDS patients [35,36], particularly sepsis-associated ARDS [37]. The murine equivalents of IL-8, MIP-2 and KC, have been reported to be the two most crucial chemokines for neutrophil recruitment [38]. Neutralization of MIP-2 significantly decreases neutrophil recruitment into the lung [39]. Both MIP-2 and KC bind to CXCR2 receptors, and blockade of CXCR2 attenuates neutrophil influx into the lung [40-43].

In the present study, we investigated the contribution of macrophage CD74 in MIF-induced neutrophil accumulation into the alveolar space. We showed previously that MIF has the ability to induce neutrophil accumulation [25]. These data suggest that MIF stimulates some types of cells in the alveolar space, to release MIP-2 and KC, which result in neutrophil accumulation in the alveolar space. Although the number of neutrophils in the alveolar space was less than in bacteria or bacterial component induced lung injury models, the current study showed that MIF administration can result in neutrophil accumulation (Fig. 1). While MIF has potent inflammatory and immunoregulatory properties, the mechanisms of cellular binding and activation remain to be fully elucidated. The extracellular domain of CD74 has been shown to bind MIF with high affinity and act as a surface membrane receptor [17]. Theoretically, MIF signaling is initiated after MIF binding to cell surface CD74. Recently, others have reported that expression of CD74 in mouse lung sections was predominantly localized to alveolar macrophage and type II alveolar epithelial cells [44]. Therefore we examined CD74 protein level and CD74 cell surface expression in macrophages and epithelial cells. Interestingly, we found that CD74 is expressed on the alveolar macrophage cell surface, suggesting that macrophages could respond to extracellular MIF (Fig. 3). In addition, we have shown that macrophages can release MIP-2 following MIF stimulation (Fig. 4C), and MIF-induced MIP-2 accumulation depends, at least in part, on p44/p42 MAPK signaling pathways as indicated in the specific inhibitor study in macrophages (Fig. 5).

The neutralization of MIF is a promising approach to develop new anti-inflammatory agents and treatments for diseases that involve increased MIF production or release [45,46]. In fact, an administration of neutralizing anti-MIF antibodies has proven therapeutically effective in animal models of sepsis [9] and in LPS-induced lung injury models [15,16]. X-ray crystallography studies show that ISO-1, an MIF-specific inhibitor, binds to the inflammatory active site of MIF in [27,28]. Treatment with ISO-1 has been reported to be effective in endotoxemia [27], polymicrobial sepsis [24] and autoimmune diabetes models [47]. Leng and colleagues have shown that anti-CD74 antibody can block MIF-induced cell proliferation in B cells and fibroblast cells [17]. Other recent studies have demonstrated that anti-CD74 antibody blocks MIF-CD74 binding in the cell surface of gastric epithelial cells [21], and anti-CD74 antibody attenuated proliferation of prostate cancer cells [22]. Moreover, a humanized anti-CD74 monoclonal antibody (hLL1) treatment has been reported to have therapeutic potential for B-cell malignancies [48]. In our study, we have shown that anti-CD74 antibody significantly inhibited the MIF-induced p44/p42 MAPK phosphorylation and MIP-2 accumulation (Fig. 6), suggesting that the MIF-induced MIP-2 accumulation depends, at least in part, on cell surface CD74 of macrophages.

We used anti-CD74 antibody in our mouse model, and have shown that anti-CD74 antibody significantly inhibited the MIF-induced neutrophil accumulation into the alveolar space as well as accumulation of MIP-2, KC (Fig. 7). The results revealed that anti-CD74 antibody has the ability to inhibit MIF-induced neutrophil accumulation in vivo. In this study, we have instilled mixtures of either MIF and ISO-1 or MIF and anti-CD74 antibody to ensure that MIF and inhibitor are delivered to an identical segment of the lung. This is important, because we believe that the native MIF binds to the receptor (CD74) to effect the inflammatory response and that the inhibitor or antibody blocks the MIF-receptor interaction. Future studies will address the question of whether the inflammatory response induced by MIF-CD74 interactions can be attenuated by intravenous administration of ISO-1 or anti-CD74 antibody, and the effective time course of administration. We believe a combination of CD74 antibody and other anti-inflammatory agents might be beneficial for treatment of neutrophilic lung inflammation.

Anti-CD74 antibody treatment reduced the number of neutrophils compared to control at 43%. This suggests that anti-CD74 antibody had a partial inhibitory effect in MIF-induced neutrophil accumulation. The data raises the possibility that MIF could activate via other signaling pathways, not CD74 alone. Indeed, an MIF endocytotic pathway has been proposed, in which MIF is taken up by the cell and binds to Jun activation domain-binding protein (Jab1) (COP9 signalosome subunit 5; CNS5). MIF would thereby inhibit Jab1-enhanced AP-1 transcriptional activity [49]. A recent study has shown that MIF activates p44/p42 MAPK pathway rapidly and transiently in a Jab1 dependent manner [50].

## Conclusion

This study shows that CD74 is expressed on the cell surface of alveolar macrophages, and potentially contributes to MIF-induced neutrophil accumulation into the alveolar space. We have demonstrated that anti-CD74 antibody inhibits MIF-induced p44/p42 MAPK phosphorylation and MIP-2 release in macrophages. Furthermore, we have demonstrated that anti-CD74 antibody treatment inhibits MIF-induced MIP-2, KC accumulation and neutrophil accumulation in mouse model. The data suggest that MIF and its receptor CD74 may be useful targets to reduce neutrophilic lung inflammation, and acute lung injury.

## Competing interests

The authors declare that they have no competing interests.

## Authors' contributions

KT carried out the experimental work, the data analysis and drafted the manuscript. KK, HML, YZ and XL participated in the discussion of the experiments. KO provided MAPK specific inhibitors and edited the manuscript. CNM provided r-MIF and edited the manuscript. YA provided MIF specific inhibitor, ISO-1. EJM provided overall leadership to the experimental design, data analysis, and preparation of the manuscript.
